# Relationship of dairy heifer reproduction with survival to first calving, milk yield and culling risk in the first lactation

**DOI:** 10.5713/ajas.19.0474

**Published:** 2019-12-24

**Authors:** István Fodor, Zsolt Lang, László Ózsvári

**Affiliations:** 1 Department of Veterinary Forensics and Economics, University of Veterinary Medicine Budapest, Budapest, H-1078, Hungary; 2 Department of Biomathematics and Informatics, University of Veterinary Medicine Budapest, Budapest, H-1078, Hungary

**Keywords:** Dairy Cattle, Fertility, Age at First Calving, Milk Yield, Longevity

## Abstract

**Objective:**

The aim of our study was to determine the associations of heifer reproductive performance with survival up to the first calving, first-lactation milk yield, and the probability of being culled within 50 days after first calving.

**Methods:**

Data from 33 large Holstein-Friesian commercial dairy herds were gathered from the official milk recording database in Hungary. The data of heifers first inseminated between January 1, 2011 and December 31, 2014 were analyzed retrospectively, using Cox proportional hazards models, competing risks models, multivariate linear and logistic mixed-effects models.

**Results:**

Heifers (n = 35,128) with younger age at conception were more likely to remain in the herd until calving, and each additional month in age at conception increased culling risk by 5.1%. Season of birth was related to first-lactation milk yield (MY1; n = 19,931), with cows born in autumn having the highest milk production (p<0.001). The highest MY1 was achieved by heifers that first calved between 22.00 and 25.99 months of age. Heifers that calved in autumn had the highest MY1, whereas calving in summer was related to the lowest milk production (p<0.001). The risk of culling within 50 days in milk in first lactation (n = 21,225) increased along with first calving age, e.g. heifers that first calved after 30 months of age were 5.52-times more likely to be culled compared to heifers that calved before 22 months of age (p<0.001). Calving difficulty was related to higher culling risk in early lactation (p<0.001). Heifers that required caesarean section were 24.01-times more likely to leave the herd within 50 days after first calving compared to heifers that needed no assistance (p<0.001).

**Conclusion:**

Reproductive performance of replacement heifers is closely linked to longevity and milk production in dairy herds.

## INTRODUCTION

Heifer raising equals to 15% to 20% of the total cost of milk production, thereby representing the second largest share among the cost factors, following feed costs [1]. The aim of the dairy farm managers is to minimize the costs and maximize the future revenue of dairy heifers, however, modern objectives also include environmental impact and animal welfare considerations [2]. Therefore, those factors that are related to the performance of replacement heifers are relevant from an economic point of view.

Evidence has been provided about even prenatal and early postnatal factors determining future performance in dairy cattle [3,4]. Season of birth was related to first-lactation milk yield (MY1) and survival to first calving, although the results varied by geographic region, and the underlying mechanisms are not completely known [4,5]. Calfhood diseases, prepubertal and postpubertal growth rate, as well as heifer fertility have been shown to influence the performance of dairy replacements in terms of age at first calving (AFC), MY1, and longevity [6,7]. In a Spanish study, heifers that needed at least five inseminations to conceive were 52% less likely to finish their first lactation compared to those heifers that conceived at first insemination [7].

Age at first calving, which is a function of age at first service and the number of services per conception (SPC), determines the length of the non-productive period before the initiation of the first lactation [8,9]. It usually takes 1 to 1.5 lactations to cover the expenses of raising replacement heifers, therefore, both MY1 and longevity influence the pay-back period of raising costs [9]. Those heifers that do not even achieve their first calving will never generate income for the farm and represent a substantial amount of loss per animal. Results regarding the optimal AFC in terms of production and lifetime revenue are quite consistent: an AFC of 22 to 25 months is generally accepted as the optimal first calving age [2]. Maximal MY1 was achieved at a first calving age of 24 to 25 months, whereas the milk production in the first five years of life peaked at an AFC of 22 to 23 months [10]. Moreover, cows that are culled or die soon after first calving will fail to cover their raising costs, thereby leading to large losses, as well. Difficult calving is one important issue that arises from inadequate heifer raising and breeding, and increases the risk of stillbirths and the mortality rate of first-lactation cows [11]. Dystocia reduces milk production, as well, however, primiparous cows experience only limited reduction in milk yield compared to their multiparous counterparts [12].

We hypothesized that certain reproductive characteristics of dairy heifers are related to their longevity and future production. The aim of our study was to assess the associations between several reproductive characteristics of the heifers and survival to first calving, MY1, and the probability of culling within 50 days in milk (DIM) of the first lactation.

## MATERIALS AND METHODS

### Data collection

Our analyses were based on the cow-level data from 33 large Holstein-Friesian commercial dairy herds in Hungary. Herds were included in this study based on the following criteria: i) use of computerized on-farm records, ii) continuous participation in milk recording dated from at least January 1, 2011, iii) herd size above 250 cows, and iv) willingness to provide data to the authors. Median (interquartile range [IQR] in parentheses) herd size of the studied farms was 577 cows (IQR = 514), with a median 305-day milk yield of 10,073 kg (IQR = 1,108).

Data of the herds were obtained using the official milk recording database provided by the Livestock Performance Testing Ltd. (Gödöllő, Hungary). Herd, animal ID, date of birth, date of inseminations, number of inseminations, results of pregnancy diagnoses, date of first calving, calving ease, 305-day milk yield in the first lactation, culling date, and the exit code were collected. Five grades were distinguished regarding the calving ease of first calving: Grade 1 (no assistance), Grade 2 (easy calving with minor assistance), Grade 3 (difficult calving with minor technical assistance), Grade 4 (difficult calving with major technical assistance), and Grade 5 (caesarean section). Regarding the exit code, heifers were either sold (i.e. moved to another farm) or culled (i.e. slaughtered or died).

Data of heifers first inseminated between January 1, 2011, and December 31, 2014, were analyzed retrospectively. In the analysis of the life histories of the heifers, 35,128 heifers were included in the follow-up, all of which were inseminated at least once. These heifers either conceived (n = 33,091), were sold prior to conception (n = 253), or were culled prior to conception (n = 1,784) based on the results of the pregnancy diagnoses. Of those heifers that conceived, 30,770 remained in the herds until first calving, whereas 406 heifers were sold, and 1,915 heifers were culled before calving. For further analysis, another 6,992 heifers were excluded from the analyses, because neither the MY1 at the time of data collection, nor the date of culling or sales were known. Those heifers, for which we had no information about the course of calving and the sex of their calves, or either abortion or stillbirth was recorded, were also excluded (n = 2,553). The final dataset used for the analysis of the risk of culling within 50 DIM in the first lactation (CULL50) consisted of 21,225 cows. If the number of days from calving to leaving the herd was between 0 and 50, the cow was considered as culled within 50 DIM (CULL50 = yes), otherwise she remained in the herd beyond 50 DIM (CULL50 = no). Altogether, 20,239 cows that first calved between 2011 and 2014 had a record of MY1, from which 308 cows were excluded, because no information about the course of calving was provided. Therefore, the final dataset used for the analysis of factors associated with MY1 contained 19,931 cows.

### Statistical analysis

Life histories of heifers were followed from birth through conception until delivery. Concomitant events such as sales for further keeping or culling were accounted for. Survival analysis was applied to model cause-specific transition risks between event states and estimate time-dependent cumulative incidence of each event [13]. The whole life history was analyzed by two subsequent competing risks survival models [13,14]. The first one focused on follow-up from birth until conception, or sales prior to conception, or culling prior to conception. The second model started follow-up on the day of successful insemination (i.e. that resulted in conception), and terminated either with the first calving, or sales, or culling. Cause-specific transition risks were estimated using Cox proportional hazards models, that included season of birth, age at conception, season of conception, and number of inseminations (1, 2, or ≥3) as explanatory variables. Marginal Cox models were applied to account for possible clustering effects of the farms [14]. Statistical analyses were performed using the survival package [15] of the R software.

The relationships between CULL50 and the reproductive parameters of heifers were examined by multivariate mixed effects logistic regression, using the lme4 package in R software [16]. The associations between MY1 and the reproductive parameters of heifers were analyzed by multivariate mixed effects linear regression, using the lme4 package in R [16]. In both regression models, season of birth, number of services per conception as heifers, AFC category, season of first calving, calving ease at first calving, and year of first calving were included as fixed effects. Season of birth and season of first calving were determined based on the calendar months of birth and calving. Regarding the number of services per conception as heifers, three categories were set up (1, 2, and ≥3 services). Heifers were categorized based on AFC as follows: <22.00, 22.00 to 23.99, 24.00 to 25.99, 26.00 to 27.99, 28.00 to 29.99, and ≥30.00 months. Regarding calving ease, five categories were differentiated according to the five calving ease grades. Year of first calving was included in the models in order to control for its potential effect on the dependent variable. Farm was the random effect. Multiple comparisons between levels of each factor were performed by Tukey’s post-hoc test using the glht procedure from the multcomp package in R [17]. Explanatory variables were tested for collinearity using the variance inflation factor (VIF): a VIF greater than 2.5 was indicative of collinearity in this study. However, no collinearity was found in our models. Data were edited in MS Excel (Microsoft Corporation, Redmond, WA, USA). The statistical analyses were performed in R version 3. 5. 0. [18]. The level of significance was set to 0.05.

## RESULTS

The major descriptive statistics of the studied population are shown in Table 1.

### Pregnancy status and survival to first calving

The probability of conception was higher in heifers born in spring and summer compared to those born in winter (relative risk [RR] = 1.075 [95% confidence interval [CI] = 1.023 to 1.130] and RR = 1.106 [1.051 to 1.163], respectively). Heifers born in autumn were less likely to conceive compared to those born in summer (RR = 0.911 [0.844 to 0.984]). Autumn-born heifers had 18% (95% CI = 2.8 to 35.4) and 20.4% (3.8 to 39.7) higher RR of culling prior to conception compared to their winter- and spring-born counterparts, respectively. Cumulative incidences of pregnancy were the highest for cows born in spring and summer, which means that by a certain age, spring- and summer-born heifers were more likely to be pregnant than those born in autumn or winter (e.g. 87.4%, 88.1%, 85.6%, and 85.5% of the spring-, summer-, autumn- and winter-born heifers were already pregnant by 600 days of age [19.74 months], respectively).

Considering pregnant heifers only, season of birth was not related to the probability of first calving. On the other hand, the season of conception was associated with first calving; heifers conceived in summer and autumn were more likely to calve than those conceived in winter and spring (p<0.001; Table 2). Similarly, cumulative incidences of calving were the highest in cows that conceived in summer or autumn. Heifers in the oldest age group (≥18 months at conception) were less likely to calve compared to any other age group (p<0.01; Table 3). Likewise, the younger a heifer at conception, the higher the probability of survival to first calving: the chance of having calved by 290 days after conception was 94.9%, 94.6%, 94.2%, 93.9%, and 92.7% as the age at conception increased from 14 to 18 months in monthly increments.

Heifers that conceived in summer were less likely to be culled than those conceived in winter and spring (RR = 0.614 [0.409 to 0.921] and RR = 0.697 [0.492 to 0.988], respectively). The risk of culling increased with the number of services per conception; the RR of culling with an SPC of 2 vs 1, ≥3 vs 1, and ≥3 vs 2 increased by 25.2% (6.3 to 47.4), 68.5% (30.6 to 117.4), and 34.6% (12.5 to 61.0), respectively. Each additional month in the age at conception increased culling risk by 5.1% (0.8 to 9.6).

### First-lactation milk yield

Season of birth (p<0.001), services per conception as heifer (p<0.001), AFC category (p<0.001), season of first calving (p<0.001), calving ease (p<0.01), and year of first calving (p<0.001) were associated with MY1 (Table 4). First-lactation milk yield was lowest in cows born in spring, whereas the highest MY1 was found in cows born in autumn. Milk production increased with the number of services per conception as a heifer, and the lowest milk production was found in heifers that calved before 22 months of age. Those heifers that needed at least three inseminations to conceive produced 288.9 kg more milk in their first lactation, on average, compared to those heifers that conceived to first insemination (p<0.001). The highest MY1 was observed at a first calving age of 22.00 to 25.99 months. Heifers that calved in summer had the lowest MY1, whereas those that calved in autumn had the highest production in their first lactation; the difference between the milk production of these groups amounted to 329.0 kg (p<0.001). First-lactation milk yield decreased numerically as calving difficulty increased. Those heifers that needed caesarean section produced 1,427.3 kg, 1,495.5 kg, and 1,391.9 kg less milk during first lactation compared to heifers with grades 1 (p = 0.08), 2 (p = 0.06), or 3 (p = 0.09), respectively.

### Culling within 50 days in milk in first lactation

Services per conception as heifer (p = 0.04), AFC category (p<0.001), year of first calving (p<0.01), and calving ease grade at first calving (p<0.001) were associated with CULL50. Those cows that needed at least three inseminations to conceive as heifers were 24% more likely to be culled within 50 DIM compared to those cows that conceived to first service as heifers (p = 0.04, Table 5). Culling risk increased steadily along with increasing age at first calving. Heifers that calved after 30 months of age were 5.52-times more likely to be culled within 50 DIM compared to heifers that calved before 22 months of age (p<0.001). A slightly increasing trend was observed between year of first calving and CULL50. Higher calving difficulty was also related to increased CULL50. Heifers that required major technical assistance or caesarean section at first calving were 6.33-times and 24.01-times more likely to be culled within 50 DIM compared to cows that needed no assistance at calving (p<0.001). Season of birth and season of first calving were not related to CULL50 (Table 5).

## DISCUSSION

### Season of birth

In our study, season of birth was associated with conception as heifers and MY1, but not with culling after first calving. By a certain age, spring- and summer-born heifers were more likely to be in calf compared to their autumn- and winter-born counterparts. This was caused partly by the high RR of conception in heifers born between March and August, but also reflects the high culling risk in heifers born in autumn. In the UK, 3.3% of heifers were culled between 15 months of age and the time of expected calving, and infertility was the most influential cause in the background [10]. Our results agree with other studies, which found that birth season is associated with milk yield after first calving, although the results are quite variable. In Slovakia, based on the results of 32 heifer calves it was found that calves born in summer had the lowest MY1, producing 1,243 kg less compared to cows born in winter [19]. Conversely, in Belgium, winter-born cows had significantly lower MY1 compared to cows born in any other season, based on the results of 74 Holstein animals [4]. Monteiro et al [5] proposed that in utero heat stress during the last six weeks of gestation significantly reduces the chance of survival to first calving and MY1. Regarding survival in first lactation, winter- and spring-born cows had better survival compared to those born in summer and autumn, according to a large-scale Spanish study [7]. The seasonal variations in the nutritional status of the dam could not cause the differences by birth season observed in our study, similarly to the research of Van Eetvelde et al [4], because on the majority of the studied farms, animals were not grazed and were fed total mixed ration all year round.

We assume that the different results compared to other studies could be attributable to the different climatic conditions of the countries. In Hungary, the herds were in the warm continental climate (largely Dfb [cold, without dry season, warm summer], partly Dfa [cold, without dry season, hot summer], according to the Köppen-Geiger climate classification) [20]. The mean air temperature of the warmest month (July) is usually ≥22°C, whereas that of the coldest month (January) is ≤0°C. Generally, a temperature-humidity index of 68 is used as the threshold for heat stress in dairy cattle in Hungary [21]. In the period of 2000 through 2008, the mean number of heat stress days was 17, with a maximum of 51 days. Conversely, Belgium lies in an oceanic climate (Cfb [temperate, without dry season, warm summer]) with cooler summers (average temperature of the warmest month: <22°C), milder winters (average temperature of the coldest month: >0°C), and more precipitation (750 to 1,000 mm/yr) that is relatively balanced throughout the year. In Spain, the cold semi-arid (BSk [arid, steppe, cold]), hot summer Mediterranean (Csa [temperate, dry and hot summer]), and the oceanic (Cfb [temperate, without dry season, warm summer]) climates can be found, as well, which means that, except for the areas with oceanic climate, the average temperature of the warmest month is >22°C [20].

We conclude that the climatic conditions (e.g. differences in exposure to heat stress) influence the relationship of birth season with performance. Although, further research is needed to clarify the exact physiological pathways of the relationship of prenatal and early postnatal factors with future performance in dairy cattle [4,5].

### Age at conception and the number of services per conception as heifers

In our study, heifers that were older at conception were more likely to be culled prior to first calving. Those heifers that required more inseminations to conceive were more likely to be culled prior to first calving, produced more milk in first lactation, and also had higher culling risk after first calving. Most authors explain poor heifer fertility with inadequate growth rates [10], however, we did not investigate the effect of body weight on reproduction. It is possible that heifers with inferior fertility, and consequently more services per conception, were older at first calving. This could result in higher milk production, as well as increased incidence of problems in the peripartum period [8]. Our results are in agreement with those of a large study from the US, where heifer conception showed a negative genetic correlation with MY1 [22]. Other studies found no relationship or even positive correlation between heifer fertility and MY1 [23]. Heifer fertility was positively associated with survival in first lactation, e.g. those heifers that needed two inseminations to conceive had 26% lower odds of completing their first lactation (compared to those heifers that conceived to first service), and the odds of survival gradually decreased as SPC increased [7]. In that study, less fertile heifers were culled more aggressively after 100 DIM as cows, possibly indicating poor milk yield. However, our results suggest that the transition into the first lactation was not successful in heifers with inferior fertility, which were, therefore, more likely to leave the herd even in early lactation, as indicated by the higher culling rate within 50 days after first calving.

### Age at first calving

The role of first calving age in influencing other economically important traits of dairy cattle, such as milk production and longevity, has been extensively studied in the recent decades [2,8]. In our study, a first calving age of 22.00 to 23.99 months was related to the highest MY1. Calving before 22 months of age reduced milk production, whereas first calving after 26 months also resulted in the gradual decrease of MY1. Heifers that first calved before 22 months of age (i.e. conception occurred at <13 months of age) were the least likely to be culled within 50 DIM, and the probability of culling increased along with increasing AFC. Herd was a random factor in our models, moreover, further analysis of the culling data revealed that only 13.22% of the cows were sent to slaughter within one year after first calving. Therefore, we assumed that our results regarding MY1 were not significantly biased by the different culling strategies of the herds. The conclusions of the different studies are consistent: the optimal AFC is 22 to 25 months, as it yields the highest profit [2,8]. Between 1980 and 2004 the average AFC of Holstein heifers decreased from 28 to 25.5 months in the US [2]. A similar, but more rapid decrease has occurred in Hungary: between 2001 and 2017 the average AFC of Holsteins has reduced by almost three months, from 28.6 to 25.7 months, which clearly shows that farmers are intensively striving for lowering first calving age [24]. However, this must be accompanied by comprehensive changes in the management, otherwise problems will arise from reduced milk yield and increased incidence of dystocia [2,8]. Our findings are in line with previous results from the literature, e.g. in a Belgian study an AFC of <22 months reduced first-lactation milk production by 11% compared to an AFC of 22 to 26 months [25]. Bach and Ahedo [6] concluded that first calving age has little correlation with MY1 if AFC is >22 months. We drew a similar conclusion, but it must be noted, that beyond 26 months of age, we found a gradually increasing MY1. According to a study of nearly 200 thousand Irish seasonal calving dairy cows, a higher AFC is accompanied by a lower chance of survival to second calving, and also the risk of being culled sooner after first calving is increased [26]. In that study, median days after first calving to culling was 187 days longer in heifers that calved at 24 months compared to those that calved at 36 months of age.

### Season of calving

We found that summer-calving heifers had the lowest, whereas autumn-calving heifers had the highest milk yield in first lactation. First calving in summer is usually associated with the lowest MY1 compared to other calving seasons [19], however, summer and autumn were associated with the highest MY1 under Belgian circumstances [25]. It is possible that in Hungary the heat stress could contribute to the lower MY1 in heifers that calved in summer, since heat stress heavily affects high-yielding dairy cows with intensive metabolism [27]. Heifers that calved in summer in our survey spent the first, high-yielding part of the lactation in a period with high probability of heat stress. Conversely, autumn-calving heifers could experience heat stress only at the end of the lactation, when the milk production is already lower. Heat stress causes diverse physiological changes in dairy cattle: dry matter intake is reduced, activity decreases, sweating and panting can be observed, along with increased heart rate, respiratory rate, and many times, increased body temperature [28,29]. Hormonal alterations also occur, but in this regard, it is often difficult to separate the effects of reduced dry matter intake and the direct effects of heat. The long-term adaptation mechanism induces a shift in energy metabolism to decrease metabolic heat production. The negative energy balance caused by reduced dry matter intake leads to increased mobilization of glucose and muscle-derived proteins, because their biological oxidation produces less heat compared to the oxidation of fatty acids [28,29]. Therefore, blood insulin concentration increases during heat stress, which leads to the decrease of blood glucose concentration. The reduced blood glucose concentration limits lactose synthesis, therefore, milk production is diminished [28].

Our findings show that the season of first calving was not associated with the chance of culling within 50 DIM, however, heifers that calved in autumn had 10% lower culling risk within 50 days after first calving. Our results support the findings of a study from California, which found no effect of calving season on culling risk in the first lactation, however, our study focused on the first 50 days after calving instead of the entire first lactation [11]. In Spain, heifers that calved in winter and spring were 25% and 37% more likely to finish their first lactation compared to those that calved in summer [7].

In summary, we can conclude that the season of calving influences MY1, largely through the timing and extent of heat stress. Conversely, we did not find a relationship between calving season and survival in early lactation. Although, these effects may vary greatly according to the differences in the climatic conditions.

### Calving difficulty

In our study, calving difficulty was related to decreased MY1 and reduced survival up to 50 DIM. Dystocia is more prevalent in heifers compared to cows, however, the milk production losses are higher in the second and later lactations compared to the first-lactation cows [12]. In a study of more than 65 thousand cows in Iran, calving difficulty reduced first-lactation milk production by 56.6 kg [12]. Dystocia does not only increase stillbirth rate, but also impairs longevity of the cow. Ettema and Santos [11] examined the effects of calving difficulties, using a scoring system from 0 to 3, increasing scores meaning increasing calving difficulty. In their study, caesarean sections were not included. Mortality rate of first-calf heifers with a calving difficulty score of 2 and 3 was significantly higher compared to those with a score of 0 and 1 (4.9% vs 2.7%, respectively). Although, the magnitude and the duration of effects of calving difficulty are greatly influenced by herd management [30].

In conclusion, higher age at conception was detrimental to survival until first calving, and similarly, higher first calving age was related to decreased MY1 and increased risk of being culled within 50 days after first calving. As heifer reproduction is closely related to future performance of cows, farm managers should use those management practices that can reduce the age at first calving.

## IMPLICATIONS

The authors determined the associations of heifer reproduction with survival to calving, MY1, and culling risk within 50 days after first calving based on the data of 35,128 heifers from 33 commercial dairy herds. Heifers younger at conception were more likely to calve, produced more milk in first lactation, and were less likely to be culled within 50 days postpartum. Caesarean section was related to a 24-fold increase in culling risk. As heifer reproduction is closely related to future performance of cows, farm managers should use those management practices that can reduce the age at first calving.

## Figures and Tables

**Table 1:** Descriptive statistics of the studied dairy heifer population

Items	Mean	SD	n
Age at first service (months)	15.58	1.48	35,128
Conception risk at 1st service (%)	48.06	0.27	35,128
Culling rate from first insemination to first calving (%)	10.53	0.16	35,128
Age at first calving (months)	25.61	2.16	30,770
Culling rate from first calving to 50 DIM (%)	6.08	0.16	21,225
305-day milk yield in first lactation (kg)	9 676	1 835	19,931

**Table 2:** The relative risk of remaining in the herd until first calving by the season of conception in pregnant Holstein heifers (n = 33,091)

Season of conception	RR estimate	95% CI	p-value
Spring vs winter	1.001	0.936 to 1.070	1.00
Summer vs winter	1.204	1.127 to 1.287	<0.001
Autumn vs winter	1.221	1.146 to 1.302	<0.001
Summer vs spring	1.203	1.127 to 1.284	<0.001
Autumn vs spring	1.221	1.143 to 1.303	<0.001
Autumn vs summer	1.014	0.954 to 1.079	0.93

RR, relative risk; CI, confidence interval.

E.g. Heifers that conceived in summer were 1.204-times (95% CI: 1.127 to 1.287) more likely to remain in the herd until calving compared to heifers that conceived in winter.

**Table 3:** The relative risk of remaining in the herd until first calving by age at conception in pregnant Holstein heifers (n = 33,091)

Age at conception (months)	RR estimate	95% CI	p-value
14.00 to 15.99 vs <14.00	1.021	0.921 to 1.132	0.95
16.00 to 17.99 vs <14.00	0.989	0.898 to 1.089	0.99
≥18.00 vs <14.00	0.869	0.785 to 0.962	<0.01
16.00 to 17.99 vs 14.00 to 15.99	0.968	0.936 to 1.002	0.07
≥18.00 vs 14.00 to 15.99	0.851	0.796 to 0.910	<0.001
≥18.00 vs 16.00 to 17.99	0.879	0.835 to 0.926	<0.001

RR, relative risk; CI, confidence interval.

E.g. Heifers that conceived after 18 months of age were 0.869 times (95% CI: 0.785 to 0.962) less likely to remain in the herd until calving compared to heifers that conceived before 14 months of age.

**Table 4:** The differences in first-lactation milk yield according to the reproductive parameters of Holstein heifers (n = 19,931)

Items	n	Mean (kg)	95% CI	p-value
Season of birth				
Spring	3,809		Reference^c^	<0.001
Summer	5,179	+161.10^ab^	89.14 to 233.04	
Autumn	5,802	+215.74^a^	141.00 to 290.48	
Winter	5,141	+116.90^b^	45.98 to 187.82	
Services per conception as heifer				
1	10,568		Reference^c^	<0.001
2	5,189	+150.79^b^	96.05 to 205.58	
3+	4,174	+288.93^a^	219.58 to 358.40	
AFC category (months)				
<22.00	150		Reference^b^	<0.001
22.00 to 23.99	3,726	+459.24^a^	199.08 to 719.25	
24.00 to 25.99	9,641	+449.19^a^	187.13 to 711.05	
26.00 to 27.99	4,158	+375.27^ab^	107.61 to 642.65	
28.00 to 29.99	1,474	+380.53^ab^	103.13 to 657.57	
≥30.00	782	+259.07^b^	−30.73 to 548.45	
Year of first calving				
2011	1,085		Reference^bc^	<0.001
2012	6,088	−84.90^c^	−189.27 to 19.49	
2013	6,439	+39.23^b^	−65.1 to 143.56	
2014	6,319	+182.44^a^	77.32 to 287.52	
Season of first calving				
Spring	4,663		Reference^b^	<0.001
Summer	4,770	−229.12^c^	−300.04 to −158.22	
Autumn	5,111	+100.10^a^	26.85 to 173.34	
Winter	5,387	+2.20^b^	−65.25 to 69.67	
Calving ease grade at first calving				
1	6,897		Reference	<0.01
2	12,152	+68.17	−2.29 to 138.46	
3	808	−35.43	−156.98 to 86.10	
4	67	−319.52	−695.93 to 56.92	
5	7	−1,427.32	−2,580.40 to −274.09	

CI, confidence interval; AFC, age at first calving.

E.g. heifers born in summer produced 161.1 kg more milk in their first lactation compared to their spring-born counterparts (p<0.05).

a–c Different superscripts within a factor indicate significant (p<0.05) difference.

**Table 5:** The factors associated with the probability of being culled within 50 days after first calving in Holstein heifers (n = 21,225)

Items	n	Odds ratio	95% CI	p-value
Season of birth				
Spring	4,098		Reference	0.27
Summer	5,461	1.02	0.84 to 1.22	
Autumn	6,147	1.05	0.87 to 1.27	
Winter	5,519	1.17	0.98 to 1.40	
Services per conception as heifer				
1	11,105		Reference^b^	0.04
2	5,493	1.14^ab^	0.99 to 1.33	
3+	4,627	1.24^a^	1.05 to 1.48	
AFC category (months)				
<22.00	162		Reference^bc^	<0.001
22.00 to 23.99	3,957	2.10^c^	0.85 to 5.24	
24.00 to 25.99	10,104	2.53^bc^	1.01 to 6.30	
26.00 to 27.99	4,464	2.92^b^	1.16 to 7.33	
28.00 to 29.99	1,624	3.41^b^	1.34 to 8.68	
≥30	914	5.52^a^	2.16 to 14.12	
Year of first calving				
2011	1,083		Reference^ab^	<0.01
2012	6,340	1.00^b^	0.73 to 1.37	
2013	6,840	1.14^ab^	0.83 to 1.55	
2014	6,962	1.30^a^	0.95 to 1.77	
Season of first calving				
Spring	5,047		Reference	0.61
Summer	5,008	1.01	0.85 to 1.21	
Autumn	5,391	0.90	0.75 to 1.09	
Winter	5,779	0.96	0.82 to 1.14	
Calving ease grade at first calving				
1	8,001		Reference^c^	<0.001
2	12,171	1.23^c^	1.02 to 1.48	
3	956	1.86^b^	1.41 to 2.45	
4	81	6.33^a^	3.7 to 10.83	
5	16	24.01^a^	8.64 to 66.77	

CI, confidence interval; AFC, age at first calving.

E.g. heifers that calved after 30 months of age had 5.52-times higher odds of culling within 50 days in milk in their first lactation compared heifers that calved before 22 months of age (p<0.05).

a–c Different superscripts within a factor indicate significant (p<0.05) difference.
